# Supplementary material for: Objective Comparison of Auditory Profiles Using Manifold Learning and Intrinsic Measures
Source: Trends Hear. 2026 Jul 1;30:23312165261461348. doi: 10.1177/23312165261461348 (PMC13323670; doi:10.1177/23312165261461348)
Supplement: Supplemental Material - Objective comparison of auditory profiles using manifold learning and intrinsic measures [file sj-pdf-1-tia-10.1177_23312165261461348.pdf]

Statistical measures

Three intrinsic measures (i.e., Davies Bouldin score, Calinski Harabasz score, and Silhouette score) were used to assess the cluster performance of the hearing loss grades (Davies & Bouldin, 1979; Caliński & Harabasz, 1974; Rousseeuw, 1987; Elkhouly et al., 2021). The **Davies Bouldin (DB) score** of the i_{th} group was calculated by (Davies & Bouldin, 1979):

$$DB = \frac{1}{k}\sum_{i=1}^{k} \max_{i \neq j} R_{ij} \qquad \text{(S1)}$$

where R_{ij} denoted the similarity measure between the i_{th} group and the j_{th} group (i.e., the most similar group of the i_{th} group), defined as:

$$R_{ij} = \frac{s_i + s_j}{d_{ij}} \qquad \text{(S2)}$$

where s_i represented the average distance between each point of group i and the centroid of that group (i.e., cluster diameter) while d_{ij} denoted the distance between group centroids i and j. A lower Davies Bouldin score reveals a better partition. The Davies Bouldin score has a lowest possible value (i.e., zero). Moreover, the **Calinski Harabasz (CH) score**, also known as the Variance Ratio Criterion, was computed by (Caliński & Harabasz, 1974):

$$CH = \frac{tr(B_k)}{tr(W_k)} * \frac{n_E - k}{k - 1} \qquad \text{(S3)}$$

where $tr(B_k)$ and $tr(W_k)$ were the traces of between-group and within-group dispersion matrices, respectively. Caliński and Harabasz (1974) provided the

computation of tr(B_k) and tr(W_k) in details. n_E represented the size of data set E while k was the number of groups. The higher the Calinski Harabasz score is, the better separation performance the hearing loss grades exhibit. The **Silhouette Index (SI)** was developed by Rousseeuw (1987) to determine how well different groups could be separated. It varied between -1 and 1, where 1 denoted the best segregation performance, while -1 meant the worst. It was defined as:

$$SI = \frac{b - a}{max(a, b)} \tag{S4}$$

where a was the mean distance between a sample and all other samples in the same group whereas b was the mean distance between a sample and all other samples in the next nearest group. We applied the python package ‘scikit-learn’ to calculate all three intrinsic measures for the raw data sets, the data sets after PCA, and the data sets transformed after t-SNE (Pedregosa et al., 2011). Besides, we performed the ANOVA test to reveal whether there was a significant difference among groups in terms of different features. The statistical comparisons were implemented using the R packages ‘rstatix’ and ‘ggpubr’ (Kassambara, 2020; 2021). If p value < 0.05 (*), 0.01 (**), 0.001 (***), and 0.0001 (****), the test was considered as being significant, highly significant, very highly significant, and extremely significant, respectively, while if p value >= 0.05 (ns), the result was not significant, implying that there was no difference among groups.
